# Application of a WeChat-based mini-app as a patient reminder in *Helicobacter pylori* eradication: a prospective multi-center randomized controlled study

**DOI:** 10.1186/s12876-022-02614-1

**Published:** 2022-12-16

**Authors:** Kefang Sun, Yishu Chen, Zhenzhen Wang, Yi Liu, Yue Pan, Xinli Mao, Lei Xu, Chaohui Jin, Ming Chen, Chaohui Yu, Lan Li

**Affiliations:** 1 Department of Gastroenterology, The First Affiliated Hospital, Zhejiang University School of Medicine, Hangzhou, China; 2 Department of Gastroenterology, Taizhou Hospital, Zhejiang University, Linhai, China; 3 Department of Gastroenterology, Ningbo First Hospital, Ningbo, China; 4 Hithink RoyalFlush Information Network Co., Ltd., Hangzhou, China

**Keywords:** Compliance, Eradication, *Helicobacter pylori*, WeChat mini-app

## Abstract

**Background:**

To improve the eradication rate of *H. pylori*, researchers have investigated the role of WeChat-based mini-app as an electronic reminding system in *H. pylori* treatment.

**Methods:**

Subjects from three medical centers were divided into two groups. Patients in the daily mini-app-based notification system group received daily notifications via the WeChat mini-app. Patients in the control group received one-time verbal education on the first clinical visit. Both groups received a 14-day quadruple therapy to eradicate *H. pylori* infection. Eradication rate, compliance, adverse events and satisfaction were evaluated.

**Results:**

Both intention-to-treat (ITT) and per-protocol (PP) analyses were conducted. The eradication rate in the daily mini-app-based notification system group was slightly higher compared with the control group (ITT analysis: 76.70% vs. 70.73%, *p* = 0.312; PP analysis: 85.87% vs. 82.86%, *p* = 0.562). The compliance was significantly higher in the daily mini-app-based notification system group (ITT analysis: 85.52% vs. 70.48%, *p* = 0.028; PP analysis: 92.39% vs. 81.90%, *p* = 0.030). The adverse event rates were similar between the two groups (PP analysis: 36.96% vs. 40.95%, *p* = 0.566). No significant difference in eradication rate was seen in each subgroup analysis by age, place of residence, grade of education, or endoscopic findings.

**Conclusion:**

The study showed that daily mini-app-based notification improved patient compliance but not *H. pylori* eradication rate.

*Trial registration* The research was registered in the Chinese Clinical Trial Registry (ChiCTR2000031011, 21/03/2020).

**Supplementary Information:**

The online version contains supplementary material available at 10.1186/s12876-022-02614-1.

## Background

*Helicobacter pylori* (*H. pylori*) is a major human pathogen related to a variety of diseases from chronic gastritis, peptic ulcer disease, gastric atrophy, to malignancies like gastric adenocarcinoma and gastric mucosa-associated lymphoid tissue (MALT) lymphoma [1]. According to studies, approximately 4.4 billion individuals were infected with *H. pylori* worldwide, comprising half the world population [2]. In China, *H. pylori* infection also remains a major public health concern. A meta-analysis published in 2017 estimated the prevalence of *H. pylori* infection in China to be 55.8% (95%CI: 51.8–59.9%) [3]. With no effective vaccines currently available, most infections will persist for a lifetime without treatment [4, 5].

Despite previous controversies, recent consensus suggests that all patients without competing considerations should be offered eradication therapies. Likewise, all patients who have received eradication therapies should be assessed with the treatment outcome, preferably via non-invasive methods [6]. In China, current guidelines suggest bismuth-containing quadruple therapy (PPI + bismuth + 2 antibiotics) as the main empirical therapy for *H. pylori* eradication, with a treatment duration of 10–14 days [7]. The present recommended regimens in China have eradication rates of over 90% [8–11]. These therapies, however, are complex and time-consuming, resulting in poor patient compliance. To improve compliance, physicians attempted traditional methods like pillboxes and medication calendars [12, 13]. In recent years, several researchers investigated the effects of short-message and telephone-based reminding systems on *H. pylori* eradication and patient compliance [14, 15]. However, the outcomes varied among different studies and sometimes contradicted each other. Thus, a prospective, multicenter study is necessary for further investigation.

In this study, we developed a WeChat mini-app-based notification system to remind patients to take their medications. Compared with telephone-based notification systems, a mini-app-based system decreases physician workload and causes less disturbance to the patients. Compared with short message-based systems, a “snooze button” was implanted in the mini-app, allowing patients to temporarily ignore the notification. The purpose of this multicenter prospective randomized controlled study is to investigate the effect of the mini-app-based notification system on the eradication rate of *H. pylori*. Other clinically relevant outcomes including patient compliance, adverse events rate, and patient satisfaction level were also studied.

## Methods

### Study design

This is a prospective, single-blinded, multi-center randomized controlled study, which was conducted from August 2020 to November 2021 in three medical centers in Zhejiang province, China: The First Affiliated Hospital of Zhejiang University School of Medicine, Taizhou Hospital of Zhejiang Province, and Ningbo First Hospital. The research was registered in the Chinese Clinical Trial Registry (ChiCTR2000031011, 21/03/2020). The study design was approved by the Clinical Research Ethics Committee of the First Affiliated Hospital, Zhejiang University School of Medicine (Reference Number: 2020-78). Patients enrolled in the study were provided with a written form of informed consent.

### Patient selection and randomization

A total of 355 patients from three different medical centers were included in this study. Inclusion criteria were as follows: (1) patients aged from 18 to 70; (2) confirmed *H. pylori* infection by at least one of the following tests: urea breath test, histology exam, rapid urease test, and positive bacterial culture; (3) patients who met the inclusion criteria for *H. pylori* eradication and willingly participated in the clinical study. Exclusion criteria were as follows: (1) serious major organ comorbidities (heart, lung, liver, kidney, etc.); (2) allergy to medications; (3) previous major gastric surgeries; (4) current pregnancy or breastfeeding; (5) history of alcohol or other illicit substance abuse; (6) previous *H. pylori* eradication treatment; (7) intake of antibiotics, proton pump inhibitor, acid suppressant or bismuth within 2 weeks; (8) illiterate, not owning a smartphone, or incapable of using mini-app due to other reasons.

Patients who met the above criteria were assigned into the daily mini-app-based notification system (DMN) group or the control group by a computer-generated random sequence during clinical visits. Patient information (phone number, group status, etc.) together with the random sequence was kept in an opaque envelope by an independent researcher. Physicians were blinded to patient group status. Patients were also instructed not to disclose the group status during follow-up phone-calls.

In the light of previous studies [16, 17], the expected value of eradication rate in the DMN group and in the control group was 90% and 75%, respectively. With an alpha level of 0.05 and 80% power, the minimum required sample size for each group was 97 cases. Therefore, we intended to include more than 100 patients per group.

### Mini-app and intervention

In this study, researchers developed a WeChat mini-app using JavaScript (Oracle Corporation, USA). By scanning the Quick Response code (QR code), patients in the DMN group could install the mini-app in WeChat instantly and started receiving notifications. Patients were instructed to hit the confirm button after taking the medications or hit the ignore button and the mini-app would notify the patients again in 10 min. The response rates and feedbacks were also collected. The design and use of the app were described in detail in the Additional file 1 and Additional file 2: Fig. S1, Additional file 3: Fig. S2, Additional file 4: Fig. S3. Patients in the control group were only verbally instructed to take the medications during the first clinical visit. Patients were encouraged to raise their concerns and questions in the mini-app, and the inquiries would be answered by physicians in time. Physicians would occasionally send educational materials or questionnaires to patients.

All patients received a 14-day quadruple therapy comprising esomeprazole (AstraZeneca Pharmaceutical Co., London, United Kingdom) 20 mg twice daily, colloidal bismuth pectin (Zhejiang DND Pharmaceutical Co., Ltd, Zhejiang, China) 200 mg twice daily, amoxicillin (Zhuhai United Laboratories, Co., Zhuhai, China) 1 g twice daily, and clarithromycin (Jiangsu Hengrui Medicine Co, Ltd, Jiangsu, China) 500 mg twice daily. Esomeprazole and colloidal bismuth pectin were taken 30 min before meals. Amoxicillin and clarithromycin were taken 30 min after meals. Medication brands and specifications were the same among the three different medical centers.

All patients in the study were informed about the research and willingly agreed to participate. Patients were educated about the expected common side effects, the rationale for the treatment, and the importance of completing the full therapeutic course. Medications for other chronic diseases were permitted. Supplement probiotics and antioxidize vitamins were not allowed during the treatment. Patient information including age, sex, grade of education, place of residence, comorbidity, and the endoscopic findings was also collected.

### Outcome assessment

Five days after the treatment ended, a physician would contact the patients from both groups for follow-up information. Patients from both groups were instructed to return to the clinic with the remaining drugs. The compliance rates were calculated via the pill-counting technique. Reasons for missed dosages, adverse effects, symptom improvement, and other clinically related information were also documented in a specific questionnaire. The questionnaire was developed based on the Patient-Reported Adverse Drug Event Questionnaire and the Generic Symptoms Questionnaire [18]. The questionnaire was mainly check-list based, which was believed to be more sensitive in determining adverse effects. And researchers also added open-ended questions at the end of the questionnaire to cover other adverse effects. Patients were interviewed by individual researchers to better identify the adverse events and given further advice on management of these adverse events. Patients’ group status was not revealed to the interviewer in order to eliminate inquiry bias. Then the patients were instructed to return to the hospital four weeks later for another urea breath test to evaluate the treatment outcomes. Primary outcome was measured as the eradication rate of the *H. pylori*. Secondary outcome was measured as compliance, satisfaction, or incidence of adverse events. Good compliance was defined as taking at least 90% of the prescribed medications. Patient satisfaction was evaluated using the 5-point LIKERT scale (1-very dissatisfied, 2-dissatisfied, 3-unsure, 4-satisfied, 5-very satisfied). Patients giving scores 4–5 were defined satisfied with the treatment.

### Statistical analyses

Data analysis was performed using SPSS software (IBM, V28.0.1). Both ITT and PP analyses were performed. Categorical variables were analyzed using Chi-square test or Fisher’s exact test. Continuous variables followed a normal distribution (Kolmogorov–Smirnov test) and then were analyzed using Student’s t-test. A *P* value < 0.05 was considered statistically significant.

## Results

### Baseline characteristics

**Fig. 1:**
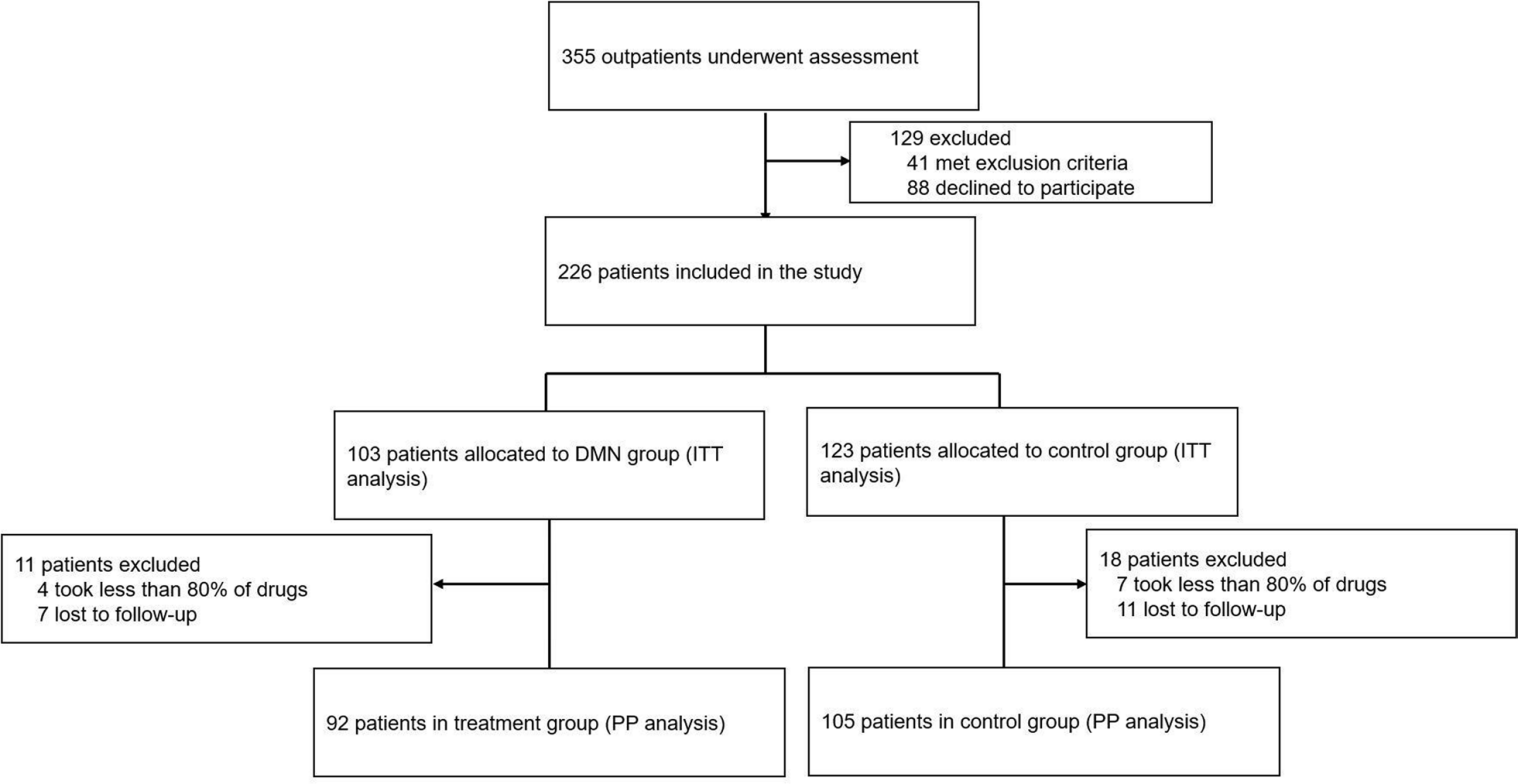
Patient inclusion and allocation

A total of 226 patients were included in this study. Patients who had taken less than 80% of the prescribed drugs, or who were lost to follow-up were included in the ITT analysis but not in the PP analysis. In the ITT analysis, 103 patients were assigned to the DMN group and 123 patients were assigned to the control group. In the PP analysis, 11 and 18 patients were excluded from the two groups respectively (Fig. 1). There were no significant differences in the patients’ baseline characteristics between two groups (Table 1).

**Table 1:** Baseline characteristics of the patients in DMN group and control group

Characteristics	DMN group (*n* = 103)	Control group (*n* = 123)	*P* value
Sex (M/F)	47/56	51/72	0.529
Age (y) (mean ± SD)	42.64 ± 12.10	44.41 ± 12.69	0.289
Grade of education			
Elementary school or less	11	16	0.608
Junior/senior high school	57	60	
College or higher	35	47	
Endoscopy findings			
Chronic gastritis	42	47	0.694
Intestinal metaplasia	6	5	0.540
Gastric/duodenal ulcer	10	15	0.553
Residence			
City	75	88	0.832
Country	28	35	
Drinking history	25	27	0.680
Smoking history	16	17	0.717
*H. pylori* infection of family members	5	6	0.780
Family history of GI malignancies	8	11	0.751

*DMN* Daily mini-app notification, *M* Male, *F* Female

### Eradication rates and compliance

In the ITT analysis, 79 out of 103 patients in the DMN group and 87 out of 123 patients in the control group eradicated *H. pylori* infection (76.70% vs. 70.73%, *p* = 0.312). In the PP analysis, the eradication rates were 85.87% and 82.86%, respectively (*p* = 0.562, Table 2).

**Table 2:** Eradication rates and compliance of DMN group and control group

	DMN group *n* (%)	Control group *n* (%)	*P* value
*Eradication rate*			
ITT analysis	79 (76.70)	87 (70.73)	0.312
PP analysis	79 (85.87)	87 (82.86)	0.562
*Compliance*			
ITT analysis	85 (82.52)	86 (70.48)	0.028
PP analysis	85 (92.39)	86 (81.90)	0.030

*DMN* Daily mini-app notification, *ITT* Intention-to-treat, *PP* Per-protocol

Proportion of patients with good compliance was significantly higher in the DMN group than in the control group (ITT analysis: 82.52% vs. 70.48%, *p* = 0.028; PP analysis: 92.39% vs. 81.90%, *p* = 0.030, Table 2).

In subgroup analysis, the study showed that patients’ baseline characteristics including age, place of residence, grade of education, and endoscopic findings had no significant influence on the effect of DMN on *H. pylori* eradication rate. It is noteworthy that compliance was significantly improved by DMN (PP analysis: 95.00% vs.77.78%, *p* = 0.023) in patients aged 40–60 (Table 3).

**Table 3:** Compliance of DMN group and control group

Compliance^a^	DMN group *n* (%)	Control group *n* (%)	*P* value
Overall	85 (92.39)	86 (81.90)	0.030
Age groups (y)			
≤ 40	41 (91.11)	38 (84.44)	0.334
40–60	38 (95.00)	35 (77.78)	0.023
> 60	6 (85.71)	13 (86.67)	1.000
Residence			
City	61 (92.42)	62 (82.67)	0.083
Country	24 (92.30)	24 (80.00)	0.352
Grade of education			
Elementary school or less	9 (90.00)	11 (91.67)	1.000
Junior/senior high school	46 (92.00)	39 (73.58)	1.000
College or higher	30 (93.75)	36 (90.00)	0.886
Endoscopic findings			
Chronic gastritis	36 (92.31)	35 (83.33)	0.374
Intestinal metaplasia	5 (83.33)	3 (60.00)	0.853
Gastric/duodenal ulcer	8 (100.00)	13 (92.86)	1.000

*DMN* Daily mini-app notification

^a^Per-protocol analysis, 92 subjects in the DMN group and 105 subjects in the control group were analyzed

### Adverse events and satisfaction

Adverse events were classified as mild-moderate (transient, well-tolerated, causing discomfort, and/or partially interfering with patients’ daily activities) or severe to unbearable, with the latter leading to discontinuation of treatment. Patients with mild-moderate adverse events were advised to continue taking medicine and closely observe their symptoms, and patients with severe adverse events were advised to discontinue treatment and seek immediate medical care. A total of 83 patients experienced adverse events. No significant difference in overall rate of adverse event was observed between the two groups (ITT analysis: 35.92% vs. 37.40%, *p* = 0.819). Among them, 77 patients experienced mild-moderate adverse effects. The most common complaints included abdominal distention (n = 22), decreased appetite (n = 21), and nausea (n = 19). There was no significant difference in mild-moderate adverse event rate between the two groups (PP analysis: 36.96% vs. 40.95%, *p* = 0.566, Table 4). Six patients experienced unbearable side effects (severe nausea, n = 3; skin rash, n = 1; severe diarrhea, n = 1; loss of appetite, n = 1), leading to medication discontinuation.

**Table 4:** Adverse events of DMN group and control group

Adverse events^a^	DMN group *n* (%)	Control group *n* (%)	*P* value
Total	34 (36.96)	43 (40.95)	0.566
Abdominal distention	9 (9.78)	13 (12.38)	0.564
Decreased appetite	9 (9.78)	12 (11.43)	0.709
Nausea	8 (8.70)	11 (10.48)	0.673
Diarrhea	4 (4.35)	3 (2.86)	0.859
Constipation	6 (6.52)	6 (5.71)	0.813
Skin rash	1 (1.09)	2 (1.90)	1.000

DMN: daily mini-app notification; ^a^Per-protocol analysis, 92 subjects in the DMN group and 105 subjects in the control group were analyzed

Patients had a higher satisfaction rate in the DMN group (PP analysis: 83.70% vs. 70.48%, *p* = 0.029, Table 5). Out of the 92 patients in the DMR group, 82 considered the mini-app helpful for the treatment.

**Table 5:** Likert scale of patient satisfaction in DMN group and control group

Likert scale^a^	DMN group *n* (%)	Control group *n* (%)
*Dissatisfied*		
1	1 (1.09)	1 (0.95)
2	1 (1.09)	3 (2.86)
3	13 (14.13)	27 (25.71)
*Satisfied*		
4	39 (42.39)	40 (38.10)
5	38 (41.30)	34 (32.38)

*DMN* Daily mini-app notification

^a^Per-protocol analysis, 92 subjects in the DMN group and 105 subjects in the control group were analyzed

## Discussion

In this study, we randomly assigned patients from three medical centers to the DMN group and the control group. For the DMN group, we asked patients to download a WeChat mini-app-based notification system, which would notify the patients to take medications on a daily basis. Patients in the control group were only offered verbal education once in outpatients. The compliance and the eventual eradication status were evaluated. We found that the DMN system significantly improved patient compliance and satisfaction rate, but rendered no significant improvement to *H. pylori* eradication rate. We also did sub-analysis in different age groups and found that in subjects aged 40–60, DMN significantly improved patient compliance. Possibly this is due to the busy lifestyle of most middle-aged people in China, which makes it more difficult for them to adhere to treatment. In this case, the mini-app served as a great reminder to them and hence had the greatest impact on compliance of this patient subgroup.

As medication compliance is crucial in treating *H. pylori* infection, researchers have utilized a variety of methods to improve compliance, which showed inconsistent results. A single-center clinical trial in 2015 showed that a daily telephone-based re-education system slightly but not significantly improved patient compliance (84.3% vs. 74.3%, *p* = 0.069) while the eradication rates were similar between the two groups (PP analysis: 62.7% vs. 71.2%, *p* = 0.230) [15]. Another study in 2018 showed that a twice-daily short-message-based re-education system increased the *H. pylori* eradication rate (PP analysis: 82.1% vs. 73.4%, *p* = 0.078) and the eradication rate was significantly higher in patients below 40 years old (PP analysis: 88.6% vs. 71.2%, *p* = 0.036) [14].

Apart from the telephone- and short-message-based programs, WeChat-based methods also showed promising potentials in improving patient compliance. WeChat is a multi-purpose instant messaging, social media, and mobile payment app developed by Tencent. By 2020, WeChat has over 1.2 billion active users, often referred to as a super-app or an app-for-everything [19]. In 2015, a prospective, multi-center study showed that using WeChat, physicians could increase the quality of patient bowel preparation. With interactive instructions delivered through WeChat, more patients attained adequate bowel preparation and lower mean total and segmental Ottawa scores [20]. Another study also showed that WeChat-based notification on the first and fourteenth days significantly improved disease-related knowledge, medication adherence, and *H. pylori* eradication rates [16]. In 2017 WeChat introduced a new feature called “mini-programs” or “mini-apps”, which allows program developers to create more basic, simplified versions of apps stored and run within WeChat. By scanning the QR code, patients can download and install the mini-apps within seconds. The development of the mini-app offered physicians a handy option to improve patient compliance.

This study was designated to take advantage of the mini-app to improve patient compliance and *H. pylori* eradication rate. Compared with a telephone-based re-education system, our method is based on an automatic system and requires less workload from physicians, causing less disturbance to patients, making it especially adaptive to a larger patient group or in busy clinical settings. By sharing the QR code, the notification system can be implanted in different medical centers instantly. In contrast to a short-message-based re-education system, mini-app is more flexible and interactive. It allows patients to temporarily ignore the notification and will automatically re-notify patients later, providing a more user-friendly experience. In our study, the mini-app also allowed researchers to monitor the patients’ behaviors, and identify those who strictly followed physicians’ instructions, further decreasing bias in the PP analysis. Furthermore, with minor alterations, the mini-app can be applied to patients with other diseases as a convenient notification tool.

Increasing antibiotic resistance has become the main reason for decreased eradication rates in recent years [3, 21]. Apart from that, poor patient adherence due to adverse events, forgetfulness, or inadequate understanding of the disease also compromises treatment outcomes [22]. In this study, the mini-app significantly improved patients’ adherence, and the adverse event rate was slightly lower in the DMN group than in the control group (36.96% vs. 40.95%) but the difference was not statistically significant. Despite increased compliance, the eradication rate was not significantly improved in the DMN group. Whether patient adherence affects the ultimate eradication rate remains controversial. Some studies showed that the *H. pylori* eradication rate would increase as a result of better patient education and compliance while some others indicated that > 60% compliance might be sufficient for *H. pylori* eradication and further improvement had no significant influence on the *H. pylori* eradication rates [21, 23, 24]. The causality among patient education, medication compliance, and clinical benefits is still worth exploring. Besides, although the study failed to demonstrate a significant improvement in the overall *H. pylori* eradication rate, patient satisfaction score was significantly higher in the DMN group, and the vast majority of patients in the DMN group believed that the mini-app helped remind them to take the medications and provided reassurance.

This study has several limitations. First, some baseline characteristics of the patients, including the history of antibiotic use and whether they lived alone or with others, were not collected. The difference of basic characteristics between the two groups might affect the eradication rate of the *H. pylori*. Second, the compliance was calculated based on the pill-counting method, which was inevitably influenced by self-report bias. Patients might forget or intentionally conceal missed dosages due to embarrassment, causing overestimation of the compliance rate. In order to reduce self-report bias, researchers encouraged patients to bring all the remaining drugs and explained to them that drug leakage was normal rather than anything to be ashamed of. Third, the follow-up rates in the DMN group and the control group were 93.2% and 91.1% respectively, and the patients lost to follow-up shared similar baseline characteristics to those included in the PP analysis, which were generally believed to be acceptable and would not cause severe distortion to the study outcomes [25, 26]. Forth, the mini-app only supported one radical cure scheme among initial treatment patients. Considering its significant impact on patient compliance, it will be applied to other treatment schemes for both initial and refractory treatment choices in future work. In addition, there might be a selection bias as the current inclusion criteria excluded illiterate patients and patients who cannot use mini-app, which unfortunately limited the general application of the mini-app. App-aided education may be achieved with the help of close relatives who were capable of using app, which will be improved in the following study.

## Conclusion

Our study showed that a mini-app-based notification system helped to improve patient adherence and satisfaction in *H. pylori* eradication, though no significant improvement was observed in the eradication rate or adverse event rate. The system provided a more efficient and flexible approach to improving patient compliance during *H. pylori* treatment in the future.

## Supplementary Information

**Additional file 1**. Supplementary methods.

**Additional file 2**. FigureS1.

**Additional file 3**. FigureS2.

**Additional file 4**. FigureS3.

## Data Availability

The data supporting the findings of this study are available from the corresponding author upon reasonable request.

## Abbreviations

DMN: Daily mini-app-based notification
ITT: Intention-to-treat
PP: Per-protocol
QR code: Quick Response code
