# Supplementary material for: Application of a WeChat-based mini-app as a patient reminder in Helicobacter pylori eradication: a prospective multi-center randomized controlled study
Source: BMC Gastroenterol. 2022 Dec 16;22:520. doi: 10.1186/s12876-022-02614-1 (PMC9756606; doi:10.1186/s12876-022-02614-1)
Supplement: Supplementary file 1 — Additional file 1. Supplementary methods. [file 12876_2022_2614_MOESM1_ESM.docx]

**Supplementary methods**

**Design and use of WeChat-based mini-app**

The mini-app was developed in WeChat, the biggest social media platform in China. Researchers used uni-app for front-end application development, and WeChat SDK to quickly collect user information and link to the patient accounts. Information management system was built through Spring MVC and React to manage drug information. The system data was stored and managed by MySQL, allowing researchers to export and analyze data under various conditions. Information collection and daily notification were achieved in a way that aligned with the users’ habits, eliminating the learning curve for new users. Patients entered information by scanning QR codes and received reminders through in-app short messages. The WeChat-based mini-app was used by following steps.

1. By scanning the QR code, a questionnaire would pop up and patients could enter their basic information (eg, name, phone number, meal time, etc.) (Supplementary Fig.1), and researchers would link users’ WeChat account to their app.
2. After patients’ WeChat accounts were linked, the mini-app would automatically start notifying patients to take the medication (Supplementary Fig.2). Patients were encouraged to raise their concerns and questions in the mini-app, and the inquiries will be answered by physicians in time.
3. Patients could temporarily ignore the notification (and the mini-app would resend the notification in 5-10 minutes) or turn off the reminder after taking the medication (Supplementary Fig.3).

**Figure legend**

Supplementary Fig.1 Input of basic information.

Supplementary Fig.2 Daily notification by Mini-app. The logos in the figure were designed by two authors (Chaohui Jin and Ming Chen).

Supplementary Fig.3 Confirm taking the medication and turn off the notification.
