# Supplementary figures and images for: Application of a WeChat-based mini-app as a patient reminder in Helicobacter pylori eradication: a prospective multi-center randomized controlled study
Source: BMC Gastroenterol. 2022 Dec 16;22:520. doi: 10.1186/s12876-022-02614-1 (PMC9756606; doi:10.1186/s12876-022-02614-1)

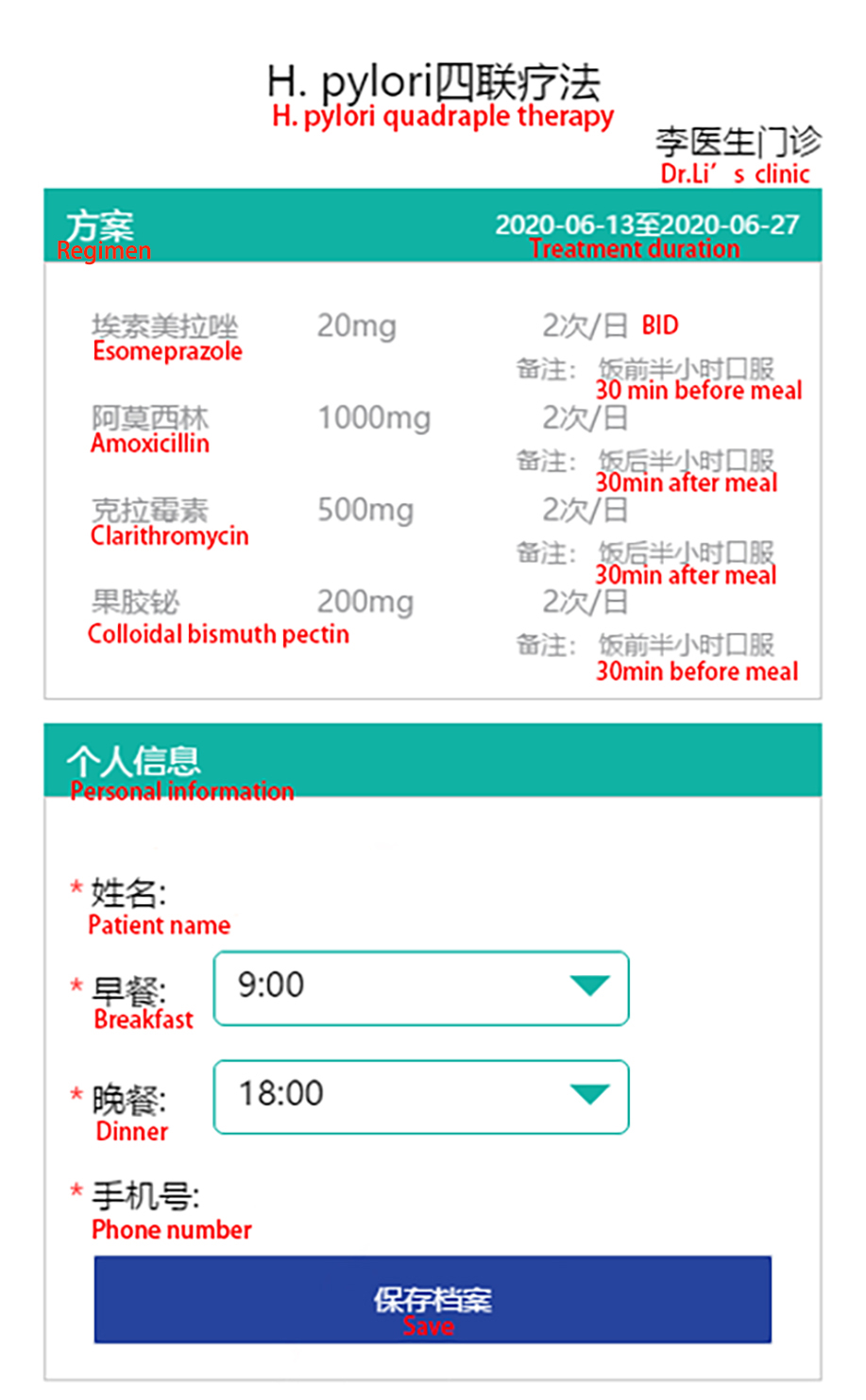

Supplement: Supplementary file 2 — Additional file 2. FigureS1. [file 12876_2022_2614_MOESM2_ESM.jpg]

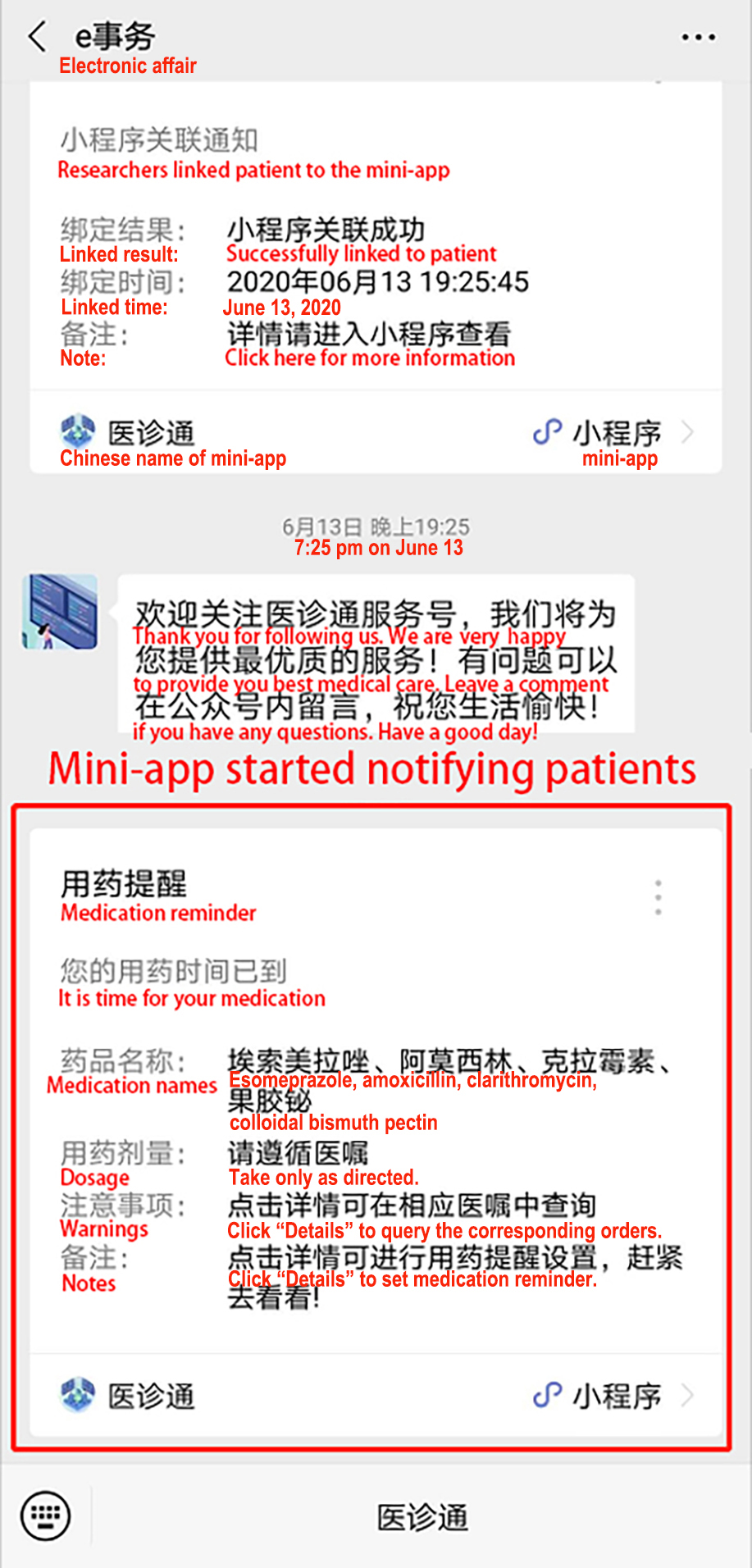

Supplement: Supplementary file 3 — Additional file 3. FigureS2. [file 12876_2022_2614_MOESM3_ESM.jpg]

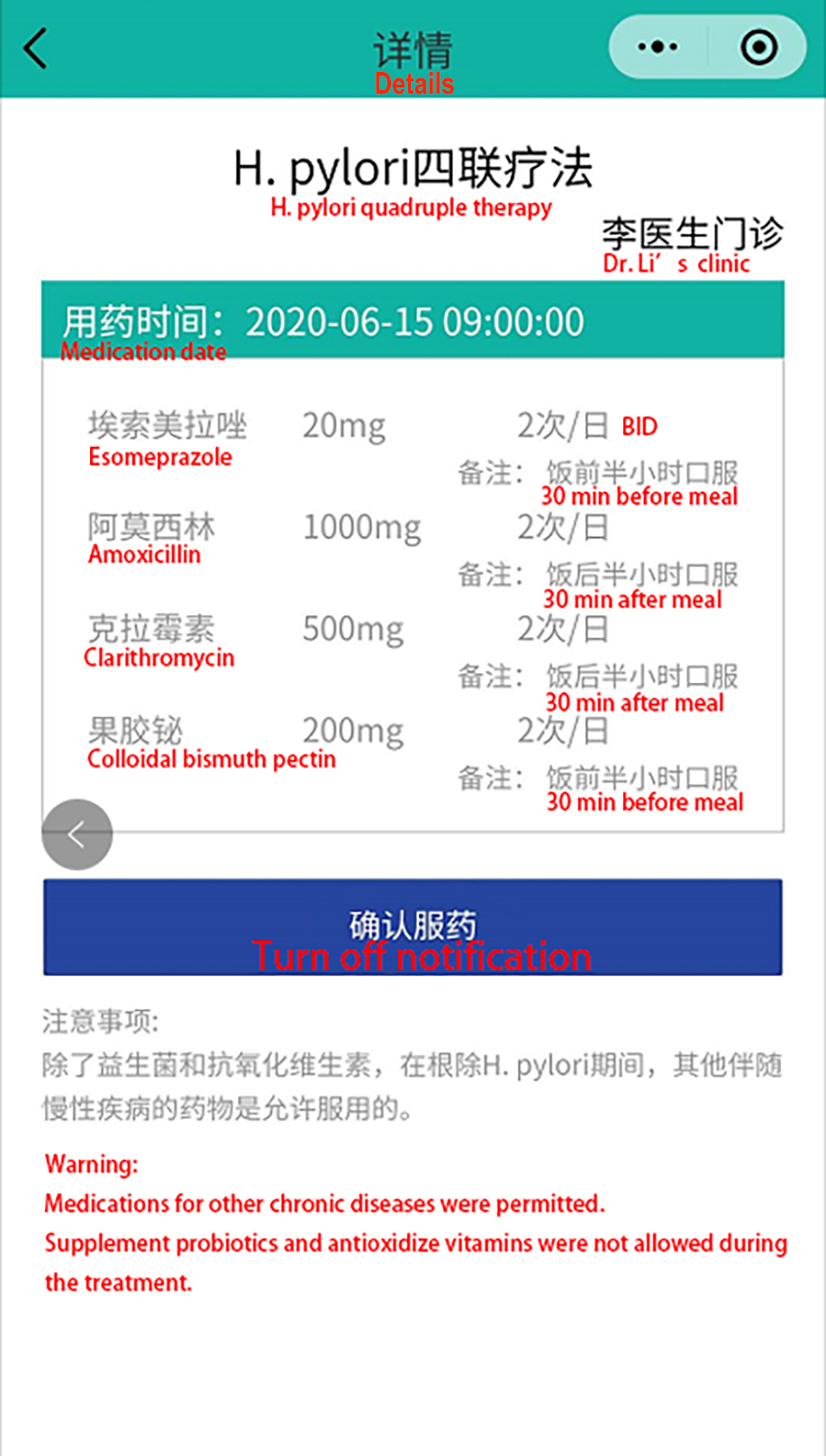

Supplement: Supplementary file 4 — Additional file 4. FigureS3. [file 12876_2022_2614_MOESM4_ESM.jpg]
